# Longitudinal validation of the GHQ-12 and PHQ-2 in Chilean social housing populations in urban regeneration contexts

**DOI:** 10.1186/s12955-026-02488-x

**Published:** 2026-02-12

**Authors:** Gabriel González-Medina, Alejandra Vives

**Affiliations:** 1 Department of Public Health, School of Medicine, Pontificia Universidad Católica de Chile, Santiago, Chile; 2 Centre for Sustainable Urban Development, CEDEUS, Santiago, Chile

**Keywords:** Mental health, Longitudinal validation, Urban regeneration, Social housing, Measurement error

## Abstract

**Background:**

Brief questionnaires have been applied in poor urban populations to monitor their mental health. Mental health outcomes encompass diverse symptoms that differ in cause and functional impact; however, most studies combine these symptoms as if they represented a single construct. Longitudinal validation of health instruments requires understanding the psychometric properties and causal structures between measurement points to distinguish measurement error from true change before interpreting intervention effectiveness. However, the longitudinal psychometric properties of the General Health Questionnaire (GHQ-12) and Patient Health Questionnaire (PHQ-2) in Latin American social housing populations remain unknown. This study assessed the dimensionality and longitudinal psychometric properties of these instruments in Chilean populations targeted by urban regeneration interventions.

**Objective:**

To assess the longitudinal psychometric properties of the General Health Questionnaire (GHQ-12) and Patient Health Questionnaire (PHQ-2) in poor Chilean urban populations undergoing urban regeneration interventions.

**Methods:**

We analyzed two waves of mental health data (6-month intervals) from 955 residents of social housing neighborhoods in Santiago and Viña del Mar, Chile. We evaluated item-level and scale-level statistics, confirmatory factor analysis, construct validity, test-retest reliability, and longitudinal measurement invariance. Following recent advances in causal inference methodology, we examined measurement error structures via directed acyclic graph principles to understand the causal implications of psychometric findings.

**Results:**

Both instruments demonstrated good psychometric properties and construct validity. The three-factor GHQ-12 structure (dysphoria, social dysfunction, loss of confidence) showed an optimal fit (comparative fit index = 0.996, Tucker‒Lewis index = 0.992, root mean square error of approximation = 0.025). Dysphoria exhibited the strongest correlation with the PHQ-2 (*r* = 0.65) and highest temporal stability (0.60), whereas social dysfunction showed the lowest stability (0.48), suggesting differential sensitivity to environmental interventions. Evidence of a response shift emerged: while configural and metric invariance held across time, scalar invariance was violated (change in comparative fit index = 0.105), indicating systematic changes in item thresholds rather than true mental health changes.

**Conclusions:**

The GHQ-12 and PHQ-2 are reliable instruments for longitudinal mental health assessment in poor urban populations. However, scalar non-invariance suggests that residents recalibrate their mental health standards while urban regeneration begins, which has important implications for interpreting intervention effects. Future studies should incorporate measurement invariance testing and latent variable approaches when evaluating complex environmental interventions.

**Supplementary information:**

The online version contains supplementary material available at 10.1186/s12955-026-02488-x.

## Background

Mental health problems, such as depression and anxiety, have become a leading cause of global disease burden and high economic implications, particularly among urban populations [1, 2]. Place of residence, a social determinant of mental health, is generally modifiable through policy, making area-based interventions a promising approach to improve mental health [3–5]. However, quantitative studies have shown inconsistent results regarding built environment upgrading and mental health [6–8].

Different issues may underlie weak evidence about interventions, among them the relatively unattended measurement error, particularly in mental health with psychological constructs depending on patient-reported outcome measures [8–12]. On the one hand, the causal inference literature underscores that population characteristics and time can be confounders between measurement error and observed variables [9, 13–15]. On the other hand, psychometric research has been confined to methodological discussions and has rarely been incorporated into causal epidemiological studies [16].

Three sources of measurement error are relevant for understanding heterogeneous results. First, psychometric properties may change across populations [17–20], resulting in differences due to measurement error rather than true construct differences [21, 22]. Second, construct validity issues arise when multidimensional instruments are reduced to single scores, potentially masking intervention effects on specific symptom domains [9, 15, 23–25]. Finally, cross-sectional validation is not enough to detect temporal measurement properties, where observed variations may reflect measurement inconsistency rather than true mental health changes [26–28].

Among those instruments frequently used to assess mental health in urban health research are the General Health Questionnaire (GHQ) and Patient Health Questionnaire (PHQ) [29]. The GHQ [30] is a 1972 instrument devised to measure diverse mental health symptoms, which are common among different psychiatric disorders, or general psychological distress in the past few weeks. In contrast, the PHQ-9 [31] and hence its shorter version, the PHQ-2, was specifically designed to screen for symptoms of depression (in the past two weeks) [32].

The validation evidence for the GHQ-12 and PHQ-2 derives predominantly from studies in high-income North American and Western European populations, whereas Latin American studies generally involve healthier and more educated samples, which cannot represent poor urban populations [31, 33–36]. The broad design of the GHQ has resulted in disagreement about dimensionality (mainly one to three dimensions) [37–39], and total score analyses cannot provide insights from separate dimensions, as evidenced during the COVID-19 pandemic, when social dysfunction increased while dysphoria decreased over time [40, 41]. Finally, whereas cross-sectional validation is predominant, longitudinal psychometric properties remain largely unexplored [37, 42], with only two studies assessing GHQ measurement invariance over time (i.e., to maintain factor structure), and we did not find longitudinal psychometrics studies for the PHQ-2, despite their frequent use in intervention research requiring repeated assessments [43, 44].

Psychometric properties can change across time and context within the same population, where the number of dimensions or factor loadings may shift between measurement occasions [22, 26, 28]. Self-reported health changes are particularly challenging to interpret because of response shifts, where individuals may recalibrate their perception and assessment of symptoms at different time points, change the meaning of constructs (i.e., change the number of dimensions) owing to life experiences and maturation, or alter their internal standards following interventions such as psychotherapy [28, 45]. Additionally, mental health outcomes may vary due to seasonality effects, natural light, and thermal exposure [46, 47]; however, studies evaluating whether observed temporal variations stem from measurement error are scarce compared with cross-sectional studies [36, 48]. In Chile, the evidence is focused on clinical samples and no use factorial analysis, and is cross-sectional, and not relate both questionnaires [33, 49–51].

Therefore, this study aimed to assess the longitudinal psychometric properties of the General Health Questionnaire (GHQ-12) and Patient Health Questionnaire (PHQ-2) in social housing populations undergoing urban regeneration.

## Methods

### Study context

In Latin America, more than one-third of the population lacks adequate housing [52]. Chile’s public housing policy has succeeded in terms of the number of housing units produced but with low quality [53]. Thus, the Chilean government has implemented programs to address quality deficits [54]. This study was part of the RUCAS (*Regeneración Urbana, Calidad de Vida y Salud*) project [55], which included two *villas* (neighborhoods) experiencing advanced material deterioration and undergoing an urban regeneration intervention. These are socioeconomically segregated neighborhoods located in the urban peripheries [56] of two major metropolitan Chilean cities, Santiago and Viña del Mar, located in the country’s central zone. The RUCAS Project [55] is a cohort study aimed at assessing the health impact of an urban regeneration policy in social housing neighborhoods.

### Design

This longitudinal study utilized baseline and second-wave data from each villa questionnaire. We selected waves 1 and 2 because they occurred before external events (social outbursts and the COVID-19 pandemic). Wave 1 in the Viña del Mar *villa* (BdM). occurred in the austral summer of 2018 (March 2018, when 8.4% of dwellings were affected), and the follow-up occurred in the winter of 2018 (September 2018, when 16.7% of dwellings were affected). Wave 1 in the Santiago *villa* (MB) occurred in the austral summer of 2019 (January 2019, 0.0% intervened dwellings), and the follow-up was completed by the end of that winter (September 2019, 3.6% intervened dwellings). Regarding public-space interventions, the new recreational space opened before wave 1 in BdM, whereas in MB it opened after wave 2. Thus, neither neighborhood experienced major interventions between the first two waves (study protocol details [55]).

### Sample

RUCAS uses a census strategy to recruit households at both study sites. Households with security concerns, dwellings used for nonresidential purposes, and dwellings without inhabitants were excluded from the sample. Trained interviewers administered the survey face-to-face to the homemakers of each household. We included all adults (≥18 years) who responded to both questionnaires in both waves. No participants were excluded by lifetime or current diagnosis or treatment. The baseline samples included 718 adults from MB and 238 adults from BdM. At follow-up, the response rates were 91.9% for MB and 87.4% for BdM. Only 9% of the participants in MB and 18.3% in BdM had more than 12 years of formal education. Overcrowding (according to Chilean standards, > 2.5 persons per bedroom) [57] affected 18.3% and 11.5% of households in MB and BdM, respectively.

### Variables

The sample description considered demographic variables: age in groups (based more on life stage), gender (male, female), years of education (primary, secondary, and superior: technical or professional), and villas.

The General Health Questionnaire (GHQ-12) is a twelve-item, Likert-type questionnaire with four response categories that screens common psychiatric disorder symptoms in the past few weeks. The GHQ-12 includes the following: six items are in the positive wording/absence of symptoms, and six are phrased negatively. Higher values indicate poorer general mental health. We used the Likert method to score the responses (0–1–2–3) [58] (total score range: 0–36). We used a Spanish translation with validation in Chilean samples [50, 51].

The Patient Health Questionnaire (PHQ-2) is a two-item questionnaire that screens for core symptoms of depression in the last two weeks [32]. The items are *“Little interest or pleasure in doing things”* (anhedonia) and *“feeling down, depressed, or hopeless”* (depressed mood). The answers ranged from *“Not at all”* to *“Nearly every day”.* The scores ranged from 0 to 6, with higher values representing a higher daily frequency of these symptoms. We used a Spanish translation with validation in a Chilean sample [59].

Self-rated general health: This item was measured with the following question: “*In general, you would say your health is …”* and ranged from 1 (*very bad*) to 7 (*very good*). This self-rated health question is among the most commonly used general health status measures [60].

Medical diagnosis of depression or anxiety disorder (measured at baseline): *Have you been diagnosed with any one of the following conditions by a health professional?* (*yes, no, does not know, does not answer*). A dichotomous variable indicated whether the participants had been diagnosed with depression or anxiety.

### Analyses

Data from both villas were pooled for analysis. We planned the analysis according to established recommendations for psychometric evaluation [27, 40, 61–63] to examine structural validity, internal consistency, test-retest reliability, and construct validity through known-group and convergent validity assessments. We tested the following a priori hypotheses: (1) the GHQ-12 would demonstrate a multidimensional structure with three factors (dysphoria, social dysfunction, and loss of confidence) based on Graetz’s model; (2) both instruments would show higher symptom prevalence among women, individuals with lower education, and middle-aged participants, which is consistent with established epidemiological patterns [64, 65]; (3) the GHQ-12 and PHQ-2 scores would correlate positively with each other and negatively with self-rated health, while showing higher scores among those with medical diagnoses of depression or anxiety; and (4) the dysphoria factor, containing core depressive symptoms (items 2, 5, 6, 9), would demonstrate the strongest correlations with the PHQ-2 given their shared focus on mood symptoms [66, 67]. Additionally, we evaluated the discriminative validity of the PHQ-2 ≥3 cutoff previously validated in Chilean populations [65] by examining its ability to differentiate the total and dimensional scores of the GHQ-12.

**Fig. 1:**
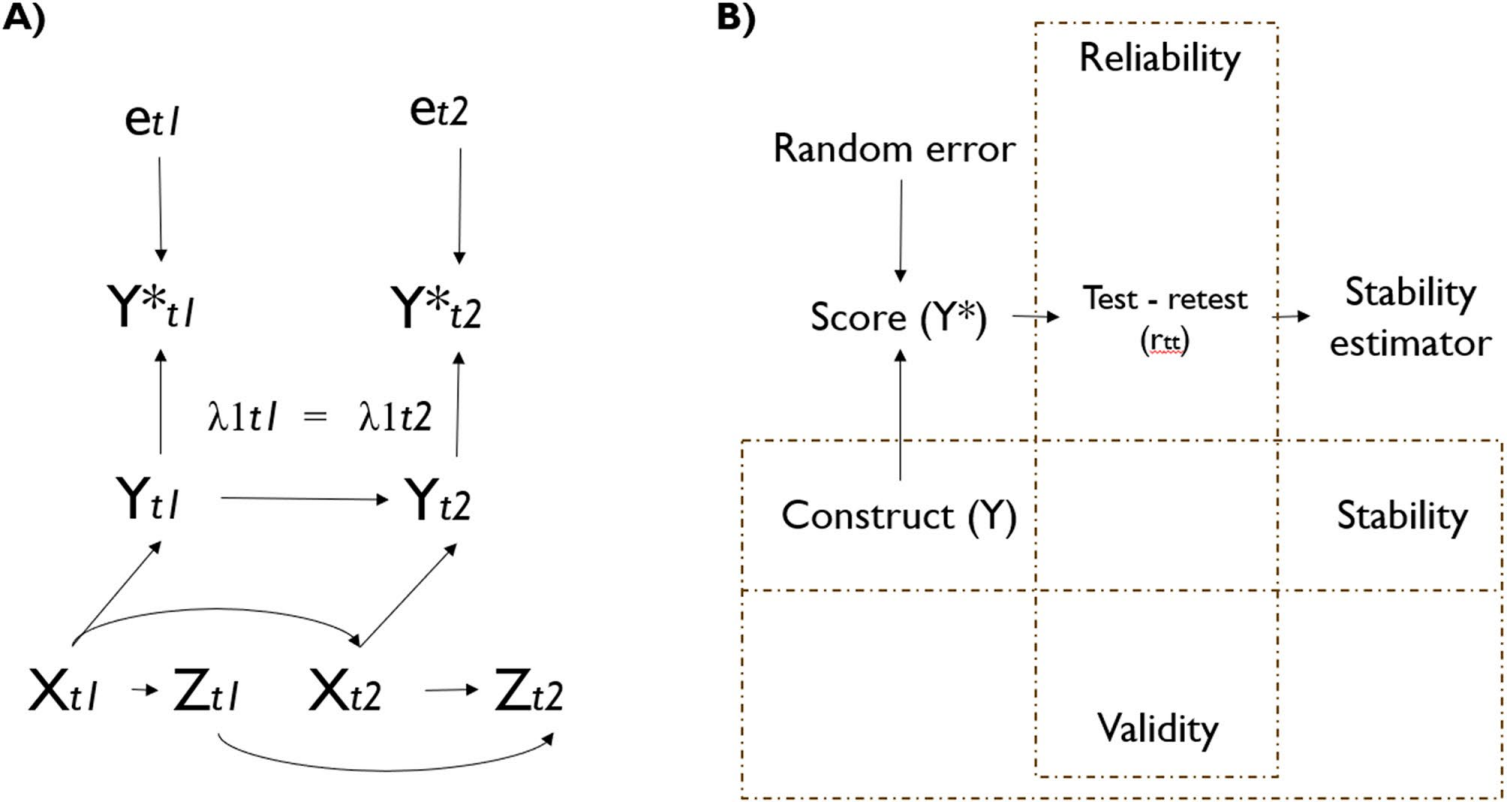
Visual summary of measurement error and psychometric concepts. Box a shows a directed acyclic graph (DAG) to represent the measurement causal structure. Y* is the score obtained from the questionnaire at time t (GHQ-12 or PHQ-2). Y is the true value of unobservable mental health outcomes (i.e., general mental health to GHQ-12 and depressive symptomatology to PHQ-2). X represents the theoretically expected associated variables with construct Y, which can be tested with known-groups and convergent analysis, whereas Z represents a variable not associated with Y. e is a random measurement error because not arrow connected this error with Y or X. The “box B” shows the conceptual link between the validity and reliability psychometric concept with the test-rest correlation, stability, and stability estimator. This illustrates that idea stability is a property of the construct (Y), whereas the stability estimator is one (but not the only) methodological approach to estimate stability, using the correlation observed variable and adjusting for internal consistency reliability

We assess longitudinal performance by examining reliability (measurement consistency), stability (construct variability), and time measurement invariance (equivalence of factor, factor loadings, and intercepts across two times) (see Fig. 1 and Appendix Methods for extended explanation). We test the following hypotheses: (1) both instruments have good test-retest reliability (0.75–0.9) [68]; (2) the resulting GHQ-12 factors, which include more stable depressive symptoms (items 10 and 11), have the highest stability [69]; and (3) the GHQ-12 has configural (equivalent factorial structure), metric (equivalent item loadings), and scalar (equivalent item intercepts) invariance within approximately 6 months from summer to winter follow-up [43].

For the longitudinal analysis, we estimated a stability value ranging from 0 to 1, where 1 indicated that no true changes between measurements were unaffected by low reliability [63]. Higher stability is expected for attributes with fewer time changes, such as personality [63]. We also tested time measurement invariance across waves, following the recommendations in the literature [70–72], where scalar invariance is required to compare the means of the observed pre- and postmeasures [72].

The statistical analysis followed four steps: 1) item-level and scale-level descriptive statistics; 2) structural validity; 3) convergent validity; and 4) test-retest reliability, stability, and measurement invariance. Step 1 describes the item and scale score distributions. Step 2 analyzes the dimensions of the GHQ-12. Step 3 analyzes the associations among the GHQ-12, PHQ-2, and relevant variables. Finally, Step 4 consisted of a longitudinal analysis of both questionnaires (see Appendix Methods).

A visual summary of the analysis is shown in Fig. 1. In “Box A,” we draw a directed acyclic graph (DAG) on the basis of the proposal of Heise (1969) [73], Hernan and Cole (2009) [14], and the measurement invariance models [22]. We then present a model of the causal hypothesis behind the psychometric test. We can then visualize how the GHQ-12 and PHQ-2 scores could change at two different moments (t) based on instrument random error (higher values indicate lower reliability). The DAG shows the causal pathways between the observed variable (questionnaire score), random measurement error (e), and unobservable variables (latent variables or constructs).

All analyses considered 95% confidence intervals and were conducted via R programming language in the R Studio integrated development environment [74]. The packages used in this study were haven, tidyverse [75], corrplot, pander, lavaan [76], and psych [77]. The RUCAS study was part of the Salurbal study (https://estudiorucas.cl/) and was approved by the Institutional Review Board of the Faculty of Medicine of Pontificia Universidad Católica de Chile (ID 170,727,004).

## Results

### Descriptive statistics

The Wave 1 participants were mostly women (81.7%), with a mean age of 48.2 years. The most common educational level was complete secondary education (52.6% and 51.5%), followed by complete primary/basic education (35.3%). Medically diagnosed depression (9.1%) was more common than anxiety disorders (5.5%). Overall, 12.1% of the patients had at least one of these diagnoses (Table 1).

**Table 1:** Sample characteristics in survey waves 1 and 2 of the RUCAS study

	Wave 1	Wave 2
	N (%)	N (%)
Sex		
Male	174 (18.2)	150 (17.2)
Female	782 (81.7)	719 (82.8)
Age		
18–25	64 (6.6)	53 (6.0)
26–35	159 (16.6)	136 (15.6)
36–45	137 (14.3)	124 (14.2)
46–59	399 (41.7)	370 (42.5)
≥60	197 (20.6)	186 (21.4)
Educational level		
Basic incomplete	62 (6.4)	59 (6.7)
Basic complete	338 (35.3)	317 (36.4)
Secondary complete	503 (52.6)	448 (51.5)
Technical–Professional	53 (5.5)	45 (5.1)
Villa*		
MB (Santiago)	718 (75.1)	661 (76.0)
BdM (Viña del Mar)	238 (24.8)	208 (23.9)
Medical diagnosis		
Depression	87 (9.1)	82(9.4)
Anxiety	55 (5.7)	50(5.7)
Depression or Anxiety	116 (12.1)	108(12.4)
Screening outcomes		
GHQ (≥5)	316 (33.0)	271 (31.0)
PHQ (≥3)	229 (23.0)	251 (28.0)

*Wave 1 (summer) data were collected in the summer of 2018 in BdM (Viña del Mar) and in the summer of 2019 in MB (Santiago). Wave 2 was the 6-month follow-up (winter)

All the GHQ-12 items had complete responses across categories (Table 2). Items 2 and 5 presented the highest means, whereas item 11 *(“Thinking of self as worthless”*) presented the lowest mean and the largest floor effect (66.7%). No ceiling effect was observed. Similarly, neither PHQ-2 item had missing values, and all response categories were used with endorsements under 0.50 and relatively high floor effects (47.9% and 45.7%, respectively). The Shapiro‒Wilk normality test revealed that the GHQ-12 and PHQ-2 scores were nonnormally distributed.

**Table 2:** Item-level descriptive statistics of GHQ-12 y PHQ-2

Items	Mean (s.d.)	Median	Skew	Kurt	Response values frequency (%)			
					0	1	2	3
GHQ-12								
1. Able to concentrate	1.23 (0.71)	1	0.42	0.22	11.6	58.5	25.2	4.6
2. Lost much sleep	1.24 (1.01)	1	0.20	−1.12	29.9	28.1	30.1	11.7
3. Playing a useful part	0.94 (0.71)	1	0.64	0.70	24.9	59.0	12.6	3.3
4. Capable of making decisions	1.01 (0.73)	1	0.54	0.40	22.0	58.1	16.2	3.5
5. Under stress	1.24(1.04)	1	0.18	−1.21	31.9	24.5	31.0	12.4
6. Could not overcome difficulties	1.04 (1.02)	1	0.44	−1.09	40.4	23.9	26.3	9.2
7. Enjoy your day-to-day activities	1.18 (0.75)	1	0.40	0.02	15.9	55.3	23.7	4.9
8. Face up to problems	1.06 (0.72)	1	0.67	0.76	18.2	61.8	15.0	4.8
9. Feeling unhappy and depressed	1.13 (1.03)	2	0.30	−1.19	36.6	23.5	29.5	10.2
10. Losing confidence	0.84 (1.01)	0	0.76	0.79	52.3	18.5	21.4	7.6
11. Thinking of self as worthless	0.56 (0.89)	0	1.37	0.64	66.3	15.7	13.2	4.6
12. Feeling reasonably happy	1.08 (0.78)	1	0.60	0.25	20.6	56.2	17.2	5.8
PHQ-2								
a. Little interest or pleasure in doing things	0.80 (0.97)	1	1.13	0.29	47.9	35.1	5.9	10.8
b. Feeling down, depressed, or hopeless	0.90 (1.04)	1	0.98	−0.28	45.7	33.5	6.1	14.5

skew: skewness. kurt: kurtosis a. 0 and 3, lowest (floor) and highest (ceiling) responses. In gray background category response with greater proportion of responses in each item

Wave 1 (summer)

All the items were positively correlated in both waves (Fig. 1. Supplementary material). Within the GHQ-12 items, the highest correlation (Spearman coefficient = 0.72) is between *“Feeling unhappy and depressed”* and *“Losing confidence”,* whereas the lowest correlation (Spearman coefficient = 0.2) is between *“Playing a useful part”* and *“Capable of making decisions”*. For the PHQ-2 items, both *“Little interest or pleasure in doing things”* and *“Feeling down, depressed, or hopeless”* had the highest correlation with the GHQ-12 item, *“Feeling unhappy and depressed”.*

### Structural validity

The results of the exploratory factor analyses for the GHQ-12 are presented in Supplementary Table 2. In the two-factor solution, Factor 1 (55% variance explained) aggregates the negatively worded items and the *“Able to concentrate”* item, whereas Factor 2 (45% proportion explained) aggregates all remaining positively worded items. In the three-factor solution, Factor 1 included Items 1, 2, 5, and 6 (31% proportion explained); Factor 2 included items 3, 4, 7, and 8 (29% proportion explained); and *“Losing confidence”* and *“Thinking of self as worthless”* were the third factors (26% proportion explained).

Table 3 shows the confirmatory factor analysis goodness-of-fit measures for the GHQ-12 based on the main models described in the literature. The Hankins model [78] showed a good fit, but Graetz’s (1991) three-factor model with Hankins’ correction had the best overall fit. These are also the only models that reach the recommended threshold for indices of fit (CFI > 0.95, TLI > 0.95, RMSEA < 0.06, SRMR < 0.08) [79].

**Table 3:** GHQ-12 model fit for main dimensional configurations described in the literature and models based on exploratory factor analysis with our data (confirmatory factor analysis, wave 1)

	N° of factors	χ^2^ (d.f)	CFI	TLI	RMSEA	SRMR
Banks et al., 1980	1	744.810 (54)	0.791	0.744	0.116	0.070
Hankins, 2008	1	150.517 (39)	**0.977**	**0.961**	**0.055**	**0.028**
Andrich and Van Schoubroeck, 1989	2	394.603 (43)	0.885	0.852	0.093	0.053
Politi et al., 1994	2	739.401 (53)	0.792	0.741	0.117	0.071
Smith, et al., 2010	2	474.878 (43)	0.844	0.801	0.103	0.060
Graetz, 1991	3	417.307 (50)	0.889	0.856	0.087	0.050
Graetz, 1991, with Hankins’s correction*	3	57.641 (36)	**0.996**	**0.992**	**0.025**	**0.028**
Rocha, 2011	3	436.523 (51)	0.883	0.849	0.089	0.051
EFA based models	2	368.632 (53)	0.876	0.845	0.091	0.058
	3	352.484 (51)	0.881	0.846	0.091	0.055

Cut-off reference values: CFI and TLI > 0.9. RMSEA < 0.6, SRMR < 0.4. CFA analysis of EFA model run using a wave 1 data of 717 cases composed of 25.3% of BdM and 68,5% of MB. *Hankin´s correction with correlated errors for negative items

The structure of the GHQ-12 is shown in Table 4, which shows the item factor loadings (λ), most of which were high ( > 0.6) [80]. The GHQ-12 dimensions (alpha and omega > 0.8) showed good internal consistency and reliability. No item dropped showed considerable improvement. Notably, the loss of confidence subdimension has good internal consistency, with only two items.

**Table 4:** Factor loadings and internal consistency reliability of the GHQ-12

	CFA		Internal consistency reliability		
	λ	**Cov**^**a**^	Alpha	Omega	Alpha if item dropped
GHQ total			0.91	0.91	
Dysphoria			0.86	0.83	
2. Lost much sleep	0.67				0.86
5. Under stress	0.75				0.82
6. Could not overcome difficulties	0.84				0.83
9. Feeling unhappy and depressed	0.87				0.82
Social dysfunction			0.85	0.82	
1. Able to concentrate	0.64				0.85
3. Playing a useful part	0.55				0.83
4. Capable of making decisions	0.64				0.82
7. Enjoy your day-to-day activities	0.81				0.81
8. Face up to problems	0.76				0.81
12. Feeling reasonably happy	0.73				0.83
Loss of confidence			0.80	0.82	
10. Losing confidence	0.89				
11. Thinking of self as worthless	0.75				
Dysphoria ~ Social dysfunction		0.79			
Dysphoria ~ Loss of confidence		0.85			
Social dysfunction ~ Loss of confidence		0.77			

λ: Factor loadings, standardized estimated by latent and observed variables. a: Covariance between latent variables. Alpha Cronbach, 1951. Omega McDonald, 1999

### Known-group, convergent, and discriminative validity

**Fig. 2:**
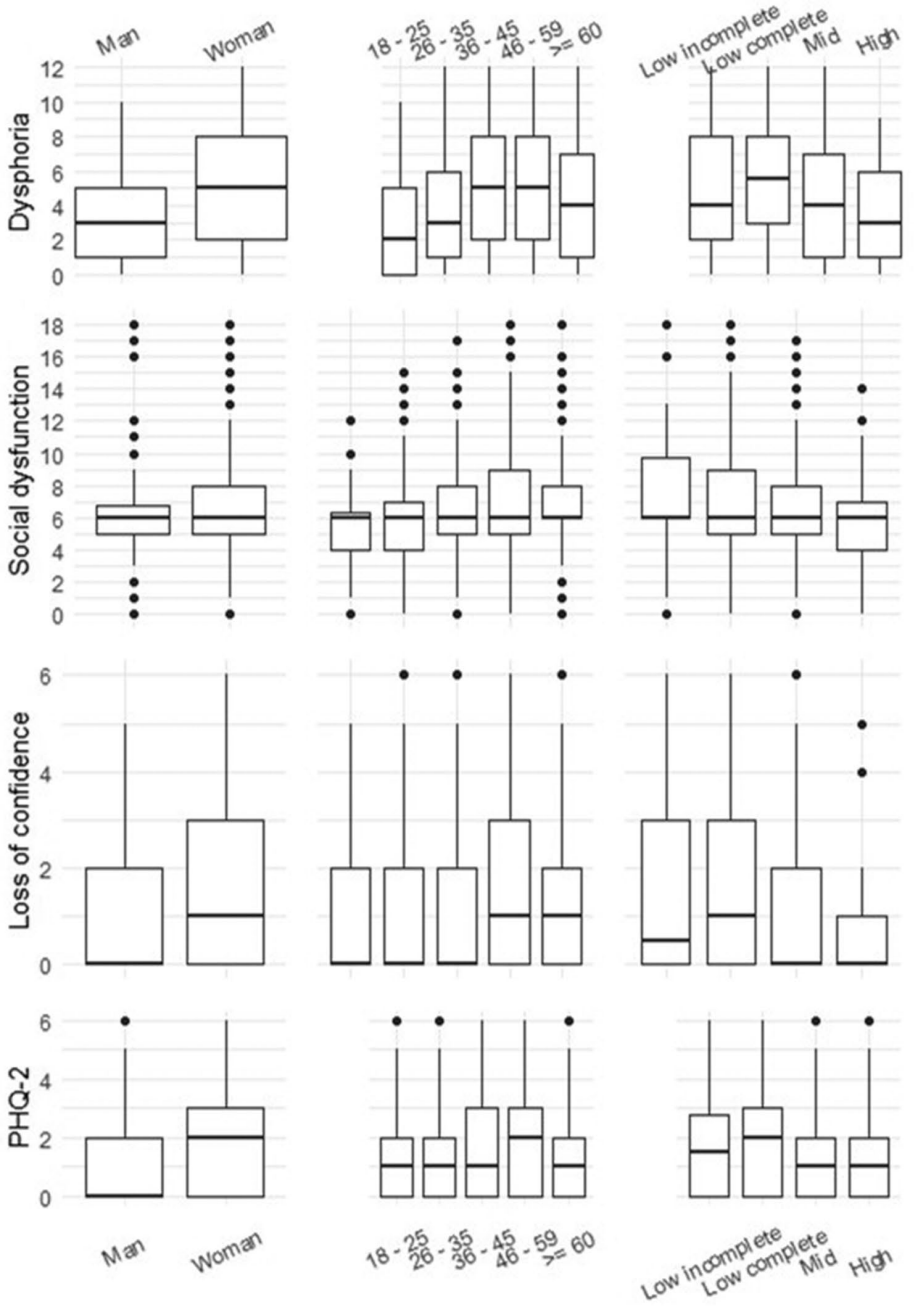
GHQ-12 and PHQ-2 distribution by known-groups (wave 1, summer). Each row shows boxplots of the three subscales of the GHQ-12 and PHQ-2 by known-groups: gender, age groups, and educational level

The total GHQ score was higher in the anxiety/depression diagnosis group (diff: 8.000049, W = 21987, *p* < 2.2e-16). Dysphoria was more common in women than in men (difference: 1.999985, W = 50194, *p* = 5.957e-08). Across age groups, there were significant differences in dysphoria (Kruskal‒Wallis χ^2^ = 43.146, df = 4, *p* value = 9.652e‒09), and Dunn (1964) Kruskal‒Wallis multiple comparisons (adjusted *p* values via the Holm method) revealed differences between the “18–34” year groups and the “36–45” and “46–59” age groups. The GHQ-12 and PHQ-2 score distributions across groups are shown in Fig. 2. Women, those with lower education, and those in the 45–59-year-old group had the highest scores on all the scales.

For convergent validity, all correlations between the GHQ-12 and PHQ-2 were statistically significant and strong. The GHQ-12 total score and each subdimension were positively correlated with the PHQ-2 score; the correlation with the PHQ-2 score was greater with the dysphoria subdimension and lowest with the loss of confidence subdimension. Perceived general health was negatively associated with both the GHQ-12 and the PHQ-2. The Mann‒Whitney Wilcoxon test indicated that subjects with a medical diagnosis of either anxiety or depression had higher GHQ-12 and PHQ-2 scores than those without a diagnosis. Finally, the PHQ-2 ≥3 cutoff in the Chilean population [65] shows excellent discriminative validity (all Cohen’s d > 0.8) [81], with participants scoring ≥3 showing approximately double the GHQ-12 distress levels compared with those scoring < 3 across both waves. All group differences were highly significant (*p* < 0.001) for all three dimensions, with large magnitude differences in both waves (see supplementary material, Table 5 s and Figures 3s and 4s).

**Table 5:** Test-retest correlations and stability for GHQ-12, its dimensions, and PHQ-2

Variables			
Wave 1 ~ Wave2	Spearman	CI 95%	Stability*
GHQ-12	0.5177	0.46 - 0.56	0.5627
Dysphoria	0.5098	0.45 - 0.56	0.5965
Social dysfunction	0.4018	0.34 - 0.46	0.4841
Loss of Confidence	0.4359	0.37 - 0.49	0.5348
PHQ- 2	0.4652	0.40 - 0.52	0.5571

Wave 1-to-6-month follow-up

The stability estimator (Röseler, 2020), where 0 is the absence of stability (construct measured completely unrelated between times) and 1 is the total stability

### Test-retest reliability, stability, and measurement invariance

Finally, all the subscales had high test-retest reliability, and stability was somewhat greater for dysphoria (0.5965) than for the GHQ-12 total (0.5627) and PHQ-2 (0.5571), whereas social dysfunction had the lowest stability (0.4841). The score in the first wave explained 34% of the variance in the dysphoria score in wave two. In contrast, only 23% of the variance in social dysfunction was explained by the wave one scores (Table 5). With respect to gender, this three-factor model showed configural, metric, and scalar measurement invariance (Table 5 in the supplementary material).

Time measurement invariance (Table 6) indicates that the scales have configural and metric invariance but not scalar invariance. Table 7 presents a comparison of intercepts. These results indicate that items are grouped into the same factors with similar factor loadings between waves but do not have the same intercepts. The intercepts were consistently greater ( > 1 on average) at the second wave.

**Table 6:** Time measurement invariance of three factor model

	χ2	df.	CFI	TLI	RMSEA	SRMR
Configural (factors)	958.353	225	0.941	0.927	0.061	0.042
Metric (loadings)	984.475	233	0.939	0.928	0.061	0.046
Scalar (intercept)	2302.954	245	0.834	0.813	0.098	0.423

Reference cutoff values: CFI and TLI > 0.9. RMSEA < 0.6, SRMR < 0.4

**Table 7:** Factor loadings and intercepts by measurement wave

Factor	Item	Factor loadings		Intercepts	
		W1	W2	W1	W2
1	2	1	1	1.24	2.3
	5	1.11	1.11	1.25	2.28
	6	1.16	1.60	1.05	2.07
	9	1.23	1.23	1.15	2.20
2	1	1.00	1.00	1.23	2.24
	3	0.95	0.95	0.95	1.94
	4	0.97	0.97	1.01	1.98
	7	1.23	1.23	1.19	2.21
	8	1.18	1.09	1.06	2.08
	12	1.16	1.16	1.09	2.03
3	10	1.00	1.00	0.86	1.85
	11	0.73	0.73	0.57	1.47

## Discussion

This study provides longitudinal (6-month follow-up, from summer to winter) evidence supporting the validity and reliability of the GHQ-12 and PHQ-2 in urban-poor populations in Chile in an urban regeneration context. For the GHQ-12, the three-factor model showed the best fit, with subscales for dysphoria, social dysfunction, and loss of confidence. The GHQ-12 and PHQ-2 were supported by known-groups, convergent, and discriminant validity in both waves. Longitudinally, the GHQ-12, its factors, and the PHQ-2 showed moderated test‒retest reliability, and the GHQ-12 structure showed time measurement invariance for factors and loadings but not scalar invariance. These findings support their applicability in urban mental health research in similar populations and contexts while considering potential response shifts.

Our study population shows patterns consistent with established evidence [64, 82–85]. We observed higher mental health symptom scores among women and middle-aged individuals. The sample exhibited moderate six-month stability coefficients (0.5 for dysphoria, 0.46 for the PHQ-2), which were somewhat lower than those reported in general population studies with similar intervals (0.7–0.8 for anxiety scales [86]); but comparable to those reported in clinical populations with negative affect scales (0.61–0.88; [27]).

Both questionnaires measured core depressive symptoms consistent with DSM-V criteria (sadness/dysphoria and anhedonia) [66]. Additionally, the PHQ-2 assesses hopelessness, a severe symptom linked to high distress and suicidality [87, 88], whereas the GHQ-12 assesses loss of confidence and worthlessness. The PHQ-2 score ≥3 cutoff demonstrated excellent discriminative validity with a modest gradient, with the strongest discrimination for the GHQ-12 total score and dysphoria and the lowest discrimination for loss of confidence (d = 1.22–1.25). In contrast, we identified clearer differences in temporal stability, with social dysfunction showing lower test‒retest reliability (*r* = 0.48) than dysphoria (*r* = 0.60), suggesting that social and functional impairments may be more context-dependent than core mood symptoms are. These findings support the three-dimensional structure of the GHQ-12 while demonstrating that the PHQ-2 serves as an effective broad-spectrum screening tool for psychological distress, which is consistent with the findings of most factor studies [89–92].

Longitudinal measurement invariance testing revealed that while the factor structure of the GHQ-12 remained stable (configural and metric invariance), participants recalibrated their response scales over the six-month period (scalar non-invariance). This pattern indicates that residents maintained consistent conceptualizations of psychological constructs but altered their internal standards for rating symptom severity [22]. Metric invariance ensures that factor loadings remain equivalent across time, validating comparative analyses of relationships between constructs, whereas scalar non-invariance necessitates caution when interpreting observed mean changes as latent psychological change [42]. Our finding corroborates previous findings [43] about item thresholds changed across 1-year and 6-year follow-ups in Finnish community samples.

The scalar non-invariance does not invalidate these instruments but rather reflects response shift—a recalibration arising from specific causal structures [28]. It suggests that people could develop new standards for mental health evaluation, through enhanced social interactions and community engagement [93], also respondents could recognize symptoms at the second measurement that they did not initially identify [45], because the process of completing questionnaires itself can increase the access to emotions and new thoughts among respondents [94], especially negative subjective experiences show an initial elevation [95, 96]. This recalibration may partially explain why studies often show inconsistent mental health benefits despite qualitative evidence of improvement [97]. Rather than indicating zero effect, the response shift represents a psychological adaptation where participants’ evolving life circumstances alter their interpretive frameworks for subjective health [28]. Although our data cannot isolate the relative contributions of intervention exposure, seasonal variation, or psychological adaptation, the preserved configural and metric invariance suggests these factors influence response scale calibration rather than construct conceptualization itself. These findings highlight the complexity of evaluating mental health, where the intervention itself may change the standards for evaluating the continuum from illness to well-being [23].

### Limitations and strengths

The primary limitation of our study was the absence of a structured psychiatric interview as the gold standard and the insufficient sample size for gender comparisons. Also, because all cases were measured first in summer, then in winter, we cannot test whether the scalar non-invariance is related to seasonal change, the 6-month interval, or the simple effect of a second measurement. A key strength of this study is the use of longitudinal data, which enhances the validity of before-and-after comparisons and allows for reliability assessment beyond the traditional but criticized Cronbach’s alpha [61, 98]. In addition, we employed EFA and CFA to test multiple supported models from the literature, enabling us to select the best-fitting model. The sample size adhered to recommendations, ensuring adequate techniques and statistical power [99–103]. This study integrates best practices in psychometric science into a causal inference perspective.

### Recommendations

Future research must recognize mental health as a multifaceted and dynamic construct that covers severe psychiatric symptoms and social well-being [104]. Thus, selecting appropriate scales and dimensions is crucial for assessing the causal mechanisms outlined in a program’s theory of change [105]. Simplifying mental health could obscure different causal pathways. For example, whereas indoor noise or temperature are causes of sleep loss [106, 107], feelings of worthlessness or the perception of not playing a useful part could require psychotherapy, community interventions, or employment support [108, 109]. In terms of bias, future studies require considering that the lack of validity produces a nonrandom measurement error [110, 111]. Thus, it is essential to identify the causal structures related to errors [14] and response shifts [111]. Analytical approaches, such as latent variable modeling or longitudinal network modeling [112], latent curve growth, and cross-lagged models [113], address time-varying intercepts (scalar invariance) and provide less biased results for mean comparisons by including a time-varying intercept. We recommend the use of DAGs [14] to evaluate potential confounders when less error-prone instruments are unavailable or multivariable latent models are unsuitable for the available data.

## Conclusions

The GHQ-12 and PHQ-2 are reliable, valid mental health measures for poor urban populations in Chile over a 6-month follow-up period from summer to winter. The GHQ-12 captures diverse mental health experiences (illness and well-being), whereas the PHQ-2, a depressive disorder screening tool, is linked to the dysphoria dimension of the GHQ-12 and is suitable for restrictive conditions such as telephone surveys. The GHQ-12 shows scalar non-invariance (response shift), indicating that studies might not yield better mental health scores, as they use higher standards for assessment. Causal analysis is needed to decide whether to use overall scores or subdimensions targeted by neighborhood interventions. Future studies and policy assessments should explore the causes of mental health response shifts in populations undergoing holistic, long-term interventions such as urban regeneration.

## Electronic supplementary material

Below is the link to the electronic supplementary material.

Supplementary material 1

## Data Availability

The data that support the findings of this study are available upon request from the author following the RUCAS, SALURBAL, and Wellcome Collaboration and Access policies. The data were not publicly available because of the sensitive nature of the questions asked in this study and the ease with which the communities could be identified. The R code used for statistical analysis is available upon request from the corresponding author.
