# Supplementary material for: Longitudinal validation of the GHQ-12 and PHQ-2 in Chilean social housing populations in urban regeneration contexts
Source: Health Qual Life Outcomes. 2026 Feb 12;24:37. doi: 10.1186/s12955-026-02488-x (PMC12997820; doi:10.1186/s12955-026-02488-x)
Supplement: Supplementary file 1 — Supplementary material 1 [file 12955_2026_2488_MOESM1_ESM.docx]

# **Supplementary**

### **Appendix methods**

In terms of bias, the lack of validity is more important than the lack of reliability, because the former produces a measurement bias (non-random measurement error), and the latter produces a random measurement error (independent of latent variables and other confounders) (Lord and Novick, 2008; Oort, Visser, and Sprangers, 2009).

In Figure 1. Y_t1_ and Y_t2_ represent the latent variable (target outcome) outcomes. The DAG implies full stability (equal to one) whenever Y_t1_ = Y_t2_ or Y*t1 = Y*t2 only if measurement error is controlled. The stability level is evidence of validity if the variation is theoretically expected, where “e_t_” represents the t-time-specific random measurement error. X is theoretically expected to be a cause of Y (e.g., concurrent validity). By contrast, Z is a specific non-casually associated variable with a construct, and the X and Z conditions provide validity evidence. The equality between factor loadings (λ) within time and Y* causally explained by the same latent variable number factor provides evidence of measurement invariance.

### 1) Item-level and scale-level statistics

First, we ran item-level and scale-level descriptive statistics to test the functioning of items and scales. We computed descriptive statistics for the GHQ-12 and PHQ-12 items: missing data, mean, standard deviation, median skewness, kurtosis, and response value frequencies to determine whether some response categories concentrated on a very high proportion of answers (>80%) [3]. A non-normal distribution (positive skewness) is expected for most items, because fewer symptoms are more frequent in the general population.

1) Construc validity

1.1) Structural validity

Second, we used Exploratory Factor analysis (EFA) to identify the number of factors and item location in the factors for the GHQ-12 using Horn’s parallel analysis [4] and oblique rotation, appropriate for correlated factors [5]. We generated two models, a two-factor model and a three-factor model, to compare the differences with a simpler model. To avoid using the same data to test the model in confirmatory factor analysis [6–8],we randomly split the sample by neighborhood into 238 (23.5% BdM neighborhood cases) for EFA and 717 (23.5% BdM neighborhood cases) for confirmatory factor analyses (CFA). We provided sensitivity analysis results of EFA by sample selection, using the data from BdM villa for EFA and MB for CFA (see supplementary tables).

We then ran a series of CFA of the GHQ-12, based on the most supported models from the literature [9], Banks et al. (1980), Schmitz et al. (1999), Graetz (1991), and Rocha et al. (2011) and the models identified in our EFA. Additionally, we added the Hankins model [10] with correlated errors in negative items, and applied that method with the best model identified [11]. We use the CFA function of R´s “lavaan” package [12]; robust statistics were estimated with WLSMV method because the variables are ordinal, and normal distribution is not assumed [13]. We reported the robust fit indices and threshold levels recommended in the literature [14] to select the best model: CFI > 0.95, TLI >0.95, RMSEA < 0.07, and SRMR < 0.08.

We estimated Omega reliability coefficients, a better estimator of reliability than Cronbach´s alpha when factor loadings are not equal [15] (we included the alpha coefficient as well to allow comparisons with previous literature): values >0.7 are considered “acceptable,” and >0.8 are considered “good” reliability for both measures [15,16].

*1.2) K*nown-group and Convergent Validity

This step tests the expected associations between the scales and other related variables [17]. First, “known-groups” hypotheses were tested as differences in scores between groups (age, gender, educational level) based on accumulated evidence and previous literature. Given non-normal distributions, we used the Mann-Whitney-Wilcoxon test for two groups and Kruskal-Wallis test for three or more groups. Second, we tested for convergent validity between the GHQ total score and its subscale scores, the PHQ-2 score, and between both and self-reported health using Spearman correlations, and between both and self-reported medical diagnosis of depression or anxiety by comparing scores across those with and without a diagnosis using the Mann-Whitney-Wilcoxon test.

*4) Test-retest reliability, stability, and measurement invariance.*

If an instrument is reliable, measurements across time will be correlated, and correlations will be higher if the construct has high stability, and no interventions have occurred [18,19]. We started with a test-retest correlation with Spearman’s correlations: GHQ-12 total and subscale scores and PHQ-2 scores in Wave 2 were correlated on their respective scales in Wave 1. We hypothesized that loss of self-confidence and worthlessness (GHQ-12 items 11 and 12) would have the highest test-retest correlation because they are associated with persistent symptoms of distress [20] and that individuals with these symptoms self-blame internally and stably [21]. Equation 1 [22] allowed us to compare stability by adjusting for the different reliabilities of the scales. The stability values range from 0 to 1, where 1 indicates no true changes between measurements that are not affected by low reliability. Higher stability is expected for attributes with fewer time changes, such as personality [22]. It is a particular approach for estimating stability, but there are other methods, such as comparing the distribution of symptoms (stability of form) [23]. In other words, stability is a construct property that is supported by theory and additional evidence, whereas reliability is a property of the measurement procedure. We used the “Equation 1” to estimate the stability for each subscale supported by confirmatory factor analysis using the following equation and the omega coefficient for internal consistency reliability.


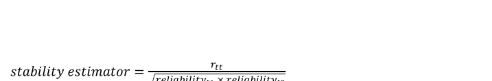


**Equation 1.** reproduced with permission from Röseler et al. (2020), and r_tt_ = measured correlation at Time 1(t_1_) and Time 2(t_2_). One indicated the highest stability, meaning no change in the measured construct, and 0 indicated the absence of stability.

Finally, we tested longitudinal measurement invariance, which means that the psychometric properties of the model were equivalent across waves. The analysis included configural (factors are equivalent), metric (factor loadings are equivalent), and scalar (intercepts are equivalent) invariance, following recommendations in the literature [24–26]. Configural and metric invariance are necessary to make inferences regarding the correlation between observed scores at different times, whereas scalar invariance is required to compare the means between observed pre- and post-measures (van de Schoot et al., 2012).

# Supplementary Tables

## Table 1s. Summary of studies comparing factor solutions to the GHQ-12.

| Authors (year) | Country | Design | Sample | | Method | Items by factors | | |
| --- | --- | --- | --- | --- | --- | --- | --- | --- |
|  |  |  | Size | Sample characteristics |  | 1 | 2 | 3 |
| Worsley & Gribbin (1977) | Australia | Cross-sectional | 603 | Women and men householders | EFA (rotation not reported | 2,5,6,7,9 | 1,3,4,8,12 | 10,11 |
| Graetz (1991) | Australia | Longitudinal | 8,998 – 6,151 | Women and men (ages 16 -25). General population | EFA (oblique rotation) | 2,5,6,9 | 1,3,4,7, 8,12 | 10,11 |
| Campbell, Walker & Farrel (2003) | Australia | Cross-sectional | 409 | Women and men (age > 16). Patients’ general surgery. | CFA | 2,5,6,7,9 | 1,3,4,8,12 | 10,11 |
| Shelvin & Adamson (2005) | Ireland | Cross-sectional | 4,633 | Women and men (mean age 47.1 years) | CFA | 2,5,6,9 | 1,3,4,7,8,12 | 10,11 |
| Rocha,et al. (2011) | Spain | Cross-sectional | 29,476 | Women and men (aged 16 -101; mean = 44 years). General population | EFA  (rotation not reported) | 2,5,9 | 1,3,4,7,8,12 | 6,10,11 |
| Rivas-Diez & Sanchez-Lopez (2013) | Chile | Cross-sectional | 371 | Women (aged 18 -72). General population | EFA (oblique rotation)  CFA | 2,5,6,9 | 1,3,4,7,8,12 | 10,11 |
| Liang, Wan, Yin (2016) | China | Cross-sectional | 525 - 526 | Women and men (aged 20-25). Civil servants. | EFA  CFA | Good fit to all type of factors models | | |

* EFA= exploratory factor analysis CFA=confirmatory factor analysis

## Figure 1s. GHQ-12 and PHQ-2 items correlations in first wave (summer).


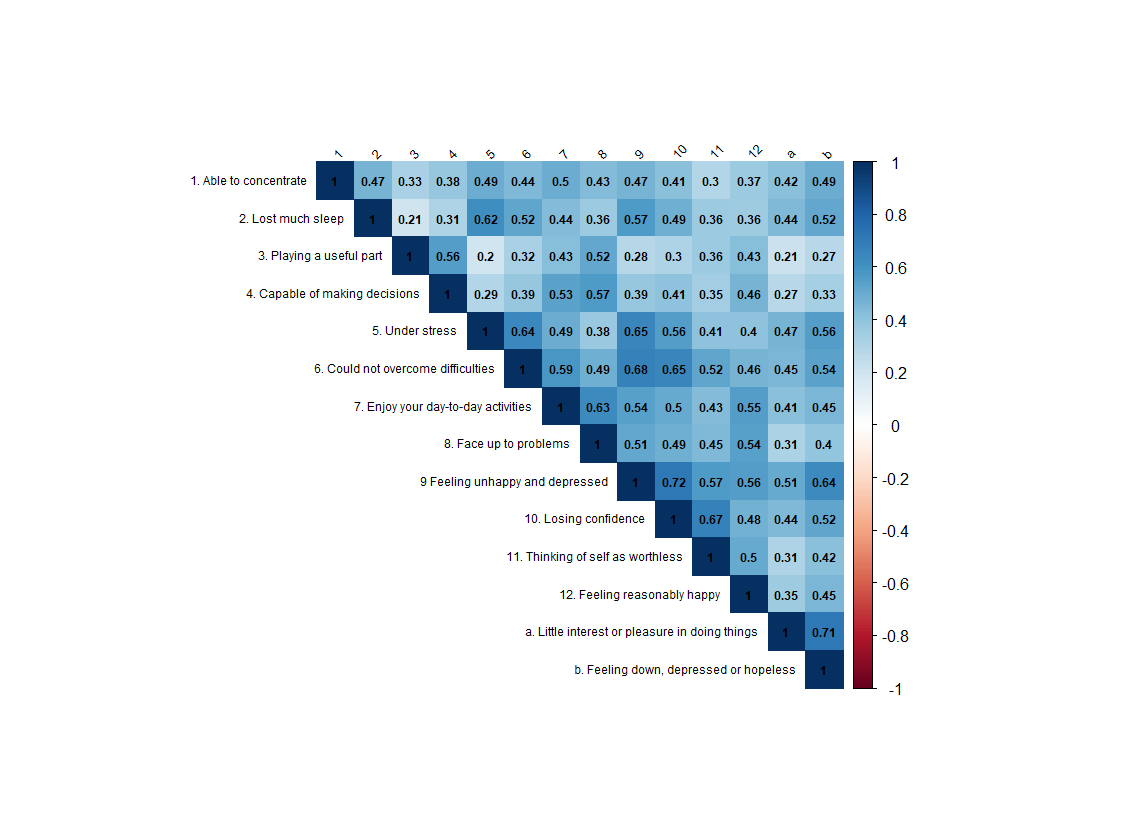


## Figure 2s. GHQ-12 and PHQ-2 items correlations in second wave (winter).


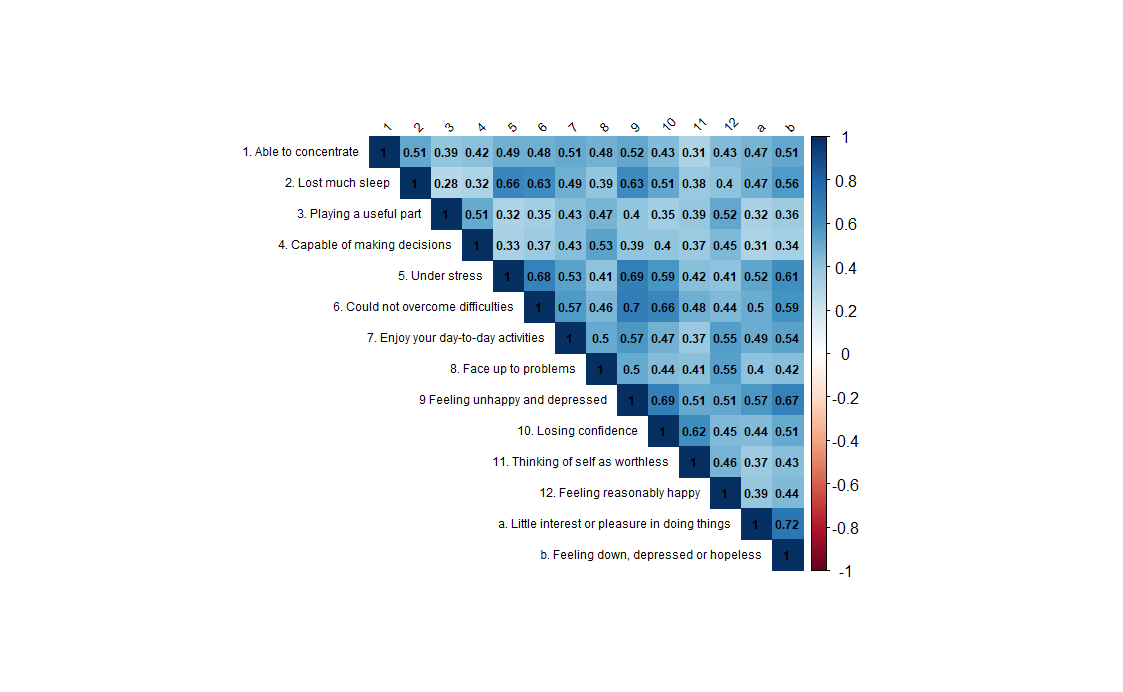


## Table 2. Item-level descriptive statistics of GHQ-12 y PHQ-2. Wave 2 (winter).

| Items | Mean (s.d.) | Median | Skew | Kurt | Response values frequency (%) | | | |
| --- | --- | --- | --- | --- | --- | --- | --- | --- |
|  |  |  |  |  | 0 | 1 | 2 | 3 |
| GHQ-12 |  |  |  |  |  |  |  |  |
| 1.Able to concentrate | 1.24 (0.68) | 1 | 0.52 | 0.44 | 9.43 | 61.3 | 24.6 | 4.60 |
| 2.Lost much sleep | 1.31 (0.99) | 1 | 0.14 | -1.04 | 25.2 | 31.3 | 30.8 | 12.6 |
| 3.Playing a useful part | 0.95 (0.63) | 1 | 0.42 | 0.79 | 21.2 | 64.3 | 12.7 | 1.61 |
| 4.Capable of making decisions | 0.99 (0.58) | 1 | 0.39 | 1.33 | 16.3 | 70.0 | 12.3 | 1.26 |
| 5.Under stress | 1.29 (0.96) | 1 | 0.10 | -1.02 | 25.3 | 30.7 | 33.6 | 10.3 |
| 6.Could not overcome difficulties | 1.08 (0.96) | 1 | 0.41 | -0.90 | 34.0 | 32.4 | 25.3 | 8.17 |
| 7.Enjoy your day-to-day activities | 1.22 (0.67) | 1 | 0.59 | 0.66 | 9.43 | 63.6 | 22.5 | 4.37 |
| 8.Face up to problems | 1.08 (0.61) | 1 | 0.67 | 1.62 | 11.9 | 70.4 | 14.9 | 2.64 |
| 9.Feeling unhappy and depressed | 1.20 (0.99) | 1 | 0.26 | -1.05 | 30.1 | 30.3 | 28.5 | 10.9 |
| 10.Losing confidence | 0.86 (0.94) | 1 | 0.77 | -0.48 | 45.5 | 29.9 | 17.9 | 6.55 |
| 11.Thinking of self as worthless | 0.47 (0.82) | 0 | 1.71 | 1.97 | 70.0 | 17.2 | 8.51 | 4.14 |
| 12.Feeling reasonably happy | 1.03 (0.66) | 1 | 0.64 | 1.21 | 16.8 | 66.1 | 13.8 | 3.22 |
|  |  |  |  |  |  |  |  |  |
| PHQ-2 |  |  |  |  |  |  |  |  |
| a. Little interest or pleasure in doing things | 0.93 (1.07) | 1 | 0.88 | -0.53 | 45.9 | 30.3 | 8.99 | 10.8 |
| b. Feeling down, depressed, or hopeless | 0.99 (1.11) | 1 | 0.79 | -0.77 | 44.5 | 29.4 | 8.63 | 17.3 |

*skew: skewness. kurt: kurtosis a. 0 and 3, lowest (floor) and highest (ceiling) responses. In gray background category response with greater proportion of responses in each item.*

## Table 2s 1. Exploratory factor analysis with wave 1 data (summer)

|  |  | Exploratory factor analysis | | | | | |
| --- | --- | --- | --- | --- | --- | --- | --- |
| Items |  | Factor 1 | Factor 2 |  | Factor 1 | Factor 2 | Factor 3 |
| 1. Able to concentrate |  | 0.57 | 0.12 |  | 0.58 | 0.22 |  |
| 2. Lost much sleep |  | 0.70 |  |  | 0.72 | 0.13 |  |
| 3. Playing a useful part |  |  | 0.85 |  |  | 0.79 |  |
| 4. Capable of making decisions |  |  | 0.73 |  |  | 0.75 |  |
| 5. Under stress |  | 0.94 | -0.16 |  | 0.83 |  |  |
| 6. Can not overcome difficulties |  | 0.74 | 0.11 |  | 0.43 |  | 0.45 |
| 7. Enjoy day-to-day activities |  | 0.35 | 0.54 |  | 0.25 | 0.43 | 0.28 |
| 8. Face up to problems |  | 0.10 | 0.74 |  |  | 0.51 | 0.39 |
| 9. Feeling unhappy and depressed |  | 0.73 | 0.16 |  | 0.33 |  | 0.63 |
| 10. Losing confidence |  | 0.63 | 0.25 |  |  |  | 0.77 |
| 11. Thinking of self as worthless |  | 0.35 | 0.49 |  |  |  | 0.83 |
| 12. Feeling reasonably happy |  | 0.27 | 0.54 |  |  | 0.27 | 0.56 |
|  |  |  |  |  |  |  |  |
|  |  |  |  |  |  |  |  |
| SS loadings |  | 3.94 | 3.22 |  | 2.41 | 2.21 | 3.08 |
| Proportion variance |  | 0.33 | 0.27 |  | 0.20 | 0.18 | 0.26 |
| Cumulative variance |  | 0.33 | 0.60 |  | 0.20 | 0.38 | 0.64 |
| Proportion explained |  | 0.55 | 0.45 |  | 0.31 | 0.29 | 0.40 |
| Cumulative proportion |  | 0.55 | 1.00 |  | 0.31 | 0.60 | 1.00 |
|  |  |  |  |  |  |  |  |

Loadings <0.1 are not presented. The larger loading weights are shaded in gray. The sample was randomly selected to include N = 238 (23.5% from Bdm and 68,5% from MB).

## Table 2s 2. Exploratory factor analysis with the BDM sample

|  |  | Exploratory factor analysis | | | | | |
| --- | --- | --- | --- | --- | --- | --- | --- |
| Items |  | Factor 1 | Factor 2 |  | Factor 1 | Factor 2 | Factor 3 |
| 1. Able to concentrate |  | 0.41 | 0.23 |  | 0.34 | 0.26 |  |
| 2. Lost much sleep |  | 0.72 |  |  | 0.64 |  |  |
| 3. Playing a useful part |  |  | 0.74 |  |  | 0.67 | 0.12 |
| 4. Capable of making decisions |  |  | 0.71 |  |  | 0.69 |  |
| 5. Under stress |  | 0.89 |  |  | 0.89 |  |  |
| 6. Can not overcome difficulties |  | 0.64 | 0.15 |  | 0.55 | 0.18 | 0.12 |
| 7. Enjoy day-to-day activities |  | 0.26 | 0.50 |  | 0.29 | 0.64 |  |
| 8. Face up to problems |  | 0.17 | 0.58 |  | 0.12 | 0.60 |  |
| 9. Feeling unhappy and depressed |  | 0.79 |  |  | 0.61 |  | 0.27 |
| 10. Losing confidence |  | 0.59 | 0.27 |  | 0.32 | 0.10 | 0.50 |
| 11. Thinking of self as worthless |  | 0.46 | 0.39 |  |  |  | 0.89 |
| 12. Feeling reasonably happy |  | 0.31 | 0.49 |  |  | 0.32 | 0.42 |
|  |  |  |  |  |  |  |  |
|  |  |  |  |  |  |  |  |
| SS loadings |  | 3.66 | 2.58 |  | 2.66 | 2.25 | 1.81 |
| Proportion Variance explained |  | 0.31 | 0.21 |  | 0.22 | 0.19 | 0.15 |
| Cumulative Variance explained |  | 0.31 | 0.52 |  | 0.22 | 0.41 | 0.56 |
| Proportion Variance explained |  | 0.59 | 0.41 |  | 0.40 | 0.33 | 0.27 |
| Cumulative Variance explained |  | 0.59 | 1.00 |  | 0.40 | 0.73 | 1.00 |
|  |  |  |  |  |  |  |  |

Loadings<0.1 are not presented. The larger loading weights are shaded in gray. N =238.

## Table 2s 3. Exploratory factor analysis with the MB sample

|  |  | Two-factor solution | |  | Three- factor solution | | |
| --- | --- | --- | --- | --- | --- | --- | --- |
| Items |  | Factor 1 | Factor 2 |  | Factor 1 | Factor 2 | Factor 3 |
| 1. Able to concentrate |  | 0.45 | 0.21 |  | 0.51 | 0.36 |  |
| 2. Lost much sleep |  | 0.73 |  |  | 0.70 |  |  |
| 3. Playing a useful part |  |  | 0.80 |  |  | 0.77 |  |
| 4. Capable of making decisions |  |  | 0.71 |  |  | 0.74 |  |
| 5. Under stress |  | 0.88 |  |  | 0.79 |  |  |
| 6. Cannot overcome difficulties |  | 0.76 | 0.10 |  | 0.49 | 0.11 | 0.34 |
| 7. Enjoy day-to-day activities |  | 0.37 | 0.49 |  | 0.30 | 0.53 |  |
| 8. Face up to problems |  | 0.17 | 0.67 |  |  | 0.65 | 0.13 |
| 9. Feeling unhappy and depressed |  | 0.79 |  |  | 0.47 |  | 0.45 |
| 10. Losing confidence |  | 0.69 | 0.16 |  | 0.28 |  | 0.66 |
| 11. Thinking of self as worthless |  | 0.38 | 0.37 |  |  | 0.15 | 0.78 |
| 12. Feeling reasonably happy |  | 0.29 | 0.48 |  |  | 0.41 | 0.33 |
|  |  |  |  |  |  |  |  |
|  |  |  |  |  |  |  |  |
| SS loadings |  | 4.05 | 2.75 |  | 2.60 | 2.68 | 2.07 |
| Proportion Variance explained |  | 0.34 | 0.23 |  | 0.22 | 0.22 | 0.17 |
| Cumulative Variance explained |  | 0.34 | 0.57 |  | 0.44 | 0.22 | 0.61 |
| Proportion Variance explained |  | 0.60 | 0.40 |  | 0.35 | 0.36 | 0.28 |
| Cumulative Variance explained |  | 0.60 | 1.00 |  | 0.35 | 0.72 | 1.00 |

Loadings<0.1 are not presented. The larger loading weights are shaded in gray. N = 717.

## Table 3s. Model fit of three exploratory factor analysis (EFA) solutions based on different samples (confirmatory factor analysis, wave 1).

| EFA sample | N° factors | χ^2^ (d.f) | CFI | TLI | RMSEA | SRMR |
| --- | --- | --- | --- | --- | --- | --- |
|  |  |  |  |  |  |  |
| Random split | 2 | 368.632 (53) | 0.876 | 0.845 | 0.091 | 0.058 |
|  | 3 | 352.484(51) | 0.881 | 0.846 | 0.091 | 0.055 |
|  |  |  |  |  |  |  |
| BdM participants | 2 | 371.283(53) | 0.895 | 0.869 | 0.092 | 0.052 |
|  | 3 | 349.750 (51) | 0.901 | 0.872 | 0.090 | 0.049 |
| MB participants | 2 | 118.154(53) | 0.802 | 0.754 | 0.072 | 0.064 |
|  | 3 | 103.494 (51) | 0.842 | 0.795 | 0.066 | 0.057 |

Random split of the 717 cases (25.3% BdM, 74,7% MB) Bdm sample = 238 and MB sample size = 717. Confirmatory factor analysis run on data for the participants not included in the corresponding EFA sample.

## Table 4s. Convergent validity tests between GHQ-12, its dimensions, PHQ-2 and self-rated health. Wave 1 (summer)

| Variables | | rho^a^ | CI 95% | Median diff.^b^ | CI 95% | W |
| --- | --- | --- | --- | --- | --- | --- |
| GHQ-12 | PHQ 2 | **0.6440** | **0.60 – 0.68** |  |  |  |
| Dysphoria | **PHQ-2** | **0.6526** | **0.61 – 0.68** |  |  |  |
| Social Dysfunction | PHQ-2 | **0.5173** | **0.46 – 0.57** |  |  |  |
| Loss of confidence | PHQ-2 | **0.5052** | **0.45 – 0.55** |  |  |  |
| GHQ-12 | Self-rated health | **-0.4034** | **0.45 – -0.34** |  |  |  |
| PHQ-2 | Self-rated health | **-****0.3979** | **-0.45 – -0.33** |  |  |  |
|  |  |  |  |  |  |  |
| GHQ-12 | Anxiety or Depression^c^ |  |  | **8.00** | **6.99 - 9.99** | 21987 |
| PHQ-2 | Anxiety or Depression^c^ |  |  | **1.99** | **1.99 - 2.00** | 22305 |

a. rho spearman correlation. b. Median difference estimated with Mann-Whitney Wilcoxon test. C Self-reported medical diagnosis of anxiety or depression (reference=no medical diagnosis). In bold significant differences (p<0.05)

## Table 5s Discriminative Validity of PHQ-2 Cut-off: GHQ-12 scores by PHQ-2 categories (summer and winter)

| Wave | PHQ-2 Category | N | GHQ-12 Total Mean (SD) | Dysphoria Mean (SD) | Social Dysfunction Mean (SD) | Loss of Confidence Mean (SD) |
| --- | --- | --- | --- | --- | --- | --- |
| 1. Summer | PHQ-2 < 3 | 726 | 10.1 (6.0) | 3.6 (3.0) | 5.6 (2.7) | 0.9 (1.4) |
|  | PHQ-2 ≥ 3 | 229 | 20.3 (7.0) | 8.1 (2.7) | 9.4 (2.7) | 2.8 (1.9) |
|  | *Cohen’s d* |  | 1.643 | 1.551 | 1.313 | 1.254 |
|  | *p-value* |  | < 0.001 | < 0.001 | < 0.001 | < 0.001 |
| 2. Winter | PHQ-2 < 3 | 619 | 9.9 (5.0) | 3.6 (2.7) | 5.5 (2.2) | 0.8 (1.2) |
|  | PHQ-2 ≥ 3 | 250 | 19.6 (6.8) | 8.0 (2.7) | 9.0 (2.7) | 2.6 (1.8) |
|  | *Cohen´s d* |  | 1.738 | 1.617 | 1.462 | 1.222 |
|  | *p-value* |  | < 0.001 | < 0.001 | < 0.001 | < 0.001 |

PHQ-2 cut-off ≥3 based on Caneo et al. (2020). All p-values from Mann-Whitney U tests. Cohen´s d effective size: 0.2 = small; 0.5 = medium; 0.8 = large, 1.4 = very large. GHQ-12 dimensions based on Graetz (1991) three-factor model: Dysphoria (items 2,5,6,9), Social Dysfunction (items 1,3,4,7,8,12), Loss of Confidence (items 10,11).

## Figure 3s. Discriminative validity GHQ-12 dimensions by PHQ-2 categories (summer)


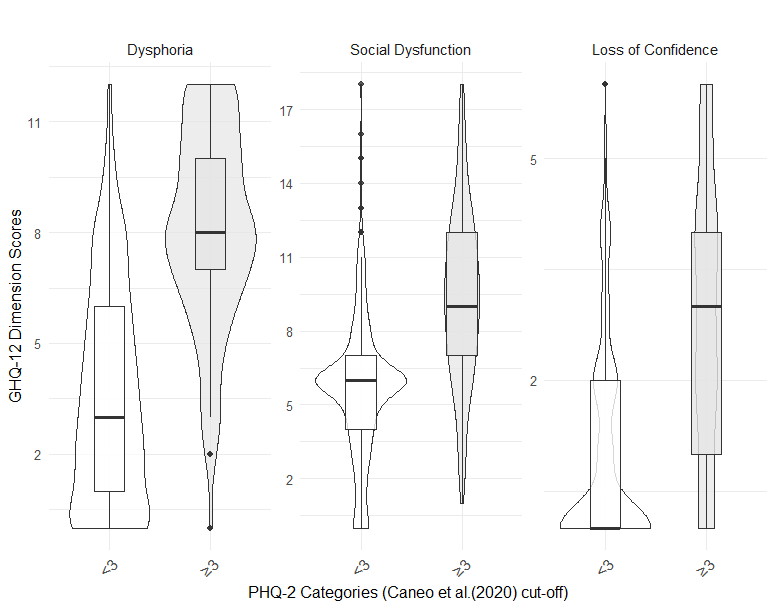


## Figure 4s. Discriminative validity GHQ-12 dimensions by PHQ-2 categories (Winter)


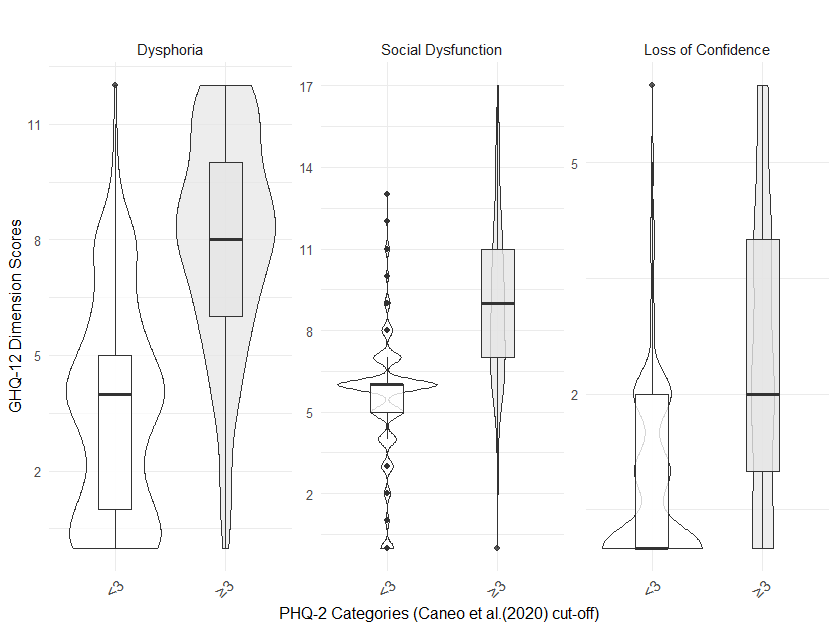


**References methods**

1. Lord F, Novick M. Statistical Theories of Mental Test Scores. U.S.A.: Information Age Publishing; 2008.

2. Oort FJ, Visser MRM, Sprangers MAG. Formal definitions of measurement bias and explanation bias clarify measurement and conceptual perspectives on response shift. J Clin Epidemiol [Internet]. 2009;62:1126–37. Available from: <http://dx.doi.org/10.1016/j.jclinepi.2009.03.013>

3. Streiner DL, Norman GR. Health Measurement Scales [Internet]. New York: Oxford University Press; 2008. Available from: <https://academic.oup.com/book/6813>

4. Horn JL. A rationale and test for the number of factors in factor analysis. Psychometrika [Internet]. 1965;30:179–85. Available from: <http://link.springer.com/10.1007/BF02289447>

5. Osborne JW. What is rotating in exploratory factor analysis? Pract Assessment, Res Eval. 2015;20:1–7.

6. Osborne JW, Fitzpatrick DC. Replication analysis in exploratory factor analysis: What it is and: Why it makes your analysis better. Pract Assessment, Res Eval. 2012;17:1–8.

7. van Prooijen JW, van der Kloot WA. Confirmatory analysis of exploratively obtained factor structures. Educ Psychol Meas. 2001;61:777–92.

8. Flora DB, Flake JK. The purpose and practice of exploratory and confirmatory factor analysis in psychological research: Decisions for scale development and validation. Can J Behav Sci. 2017;49:78–88.

9. Tomás JM, Hontangas P, Oliver A, Galiana L, Sancho P. More on the dimensionality of the GHQ-12: Competitive confirmatory models. Univ Psychol. 2019;18.

10. Hankins M. The reliability of the twelve-item general health questionnaire (GHQ-12) under realistic assumptions. BMC Public Health. 2008;8:1–7.

11. Hermida R. The Problem of Allowing Correlated Errors in Structural Equation Modeling: Concerns and Considerations. Comput Methods Soc Sci. 2015;3:5–17.

12. Rosseel Y. lavaan: An R Package for Structural Equation Modeling. J Stat Softw. 2012;48:1–36.

13. Li C-H. Confirmatory factor analysis with ordinal data: Comparing robust maximum likelihood and diagonally weighted least squares. Behav Res Methods [Internet]. 2016;48:936–49. Available from: <http://link.springer.com/10.3758/s13428-015-0619-7>

14. Efird JT, Turrini A, Boateng GO, Neilands TB, Frongillo EA, Melgar-Quiñonez HR, et al. Best Practices for Developing and Validating Scales for Health, Social, and Behavioral Research: A Primer. Front Public Heal | [www.frontiersin.org](https://www.frontiersin.org) [Internet]. 2018;1:149. Available from: [www.frontiersin.org](https://www.frontiersin.org)

15. Padilla MA, Divers J. A Comparison of Composite Reliability Estimators: Coefficient Omega Confidence Intervals in the Current Literature. Educ Psychol Meas. 2016;76:436–53.

16. Dunn TJ, Baguley T, Brunsden V. From alpha to omega: A practical solution to the pervasive problem of internal consistency estimation. Br J Psychol. 2014;105:399–412.

17. Flake JK, Pek J, Hehman E. Construct Validation in Social and Personality Research. Soc Psychol Personal Sci [Internet]. 2017;8:370–8. Available from: <http://journals.sagepub.com/doi/10.1177/1948550617693063>

18. Cronbach LJ, Meehl PE. Construct validity in psychological tests. Psychol Bull. 1955;52:281–302.

19. Peter JP. Reliability: A Review of Psychometric Basics and Recent Marketing Practices. J Mark Res [Internet]. 1979;16:6. Available from: <https://www.jstor.org/stable/3150868?origin=crossref>

20. Jokela M, García-Velázquez R, Komulainen K, Savelieva K, Airaksinen J, Gluschkoff K. Specific symptoms of the General Health Questionnaire (GHQ) in predicting persistence of psychological distress: Data from two prospective cohort studies. J Psychiatr Res. 2021;143:550–5.

21. Harrison P, Lawrence AJ, Wang S, Liu S, Xie G, Yang X, et al. The Psychopathology of Worthlessness in Depression. Front Psychiatry. 2022;13:1–9.

22. Röseler L, Wolf D, Leder J, Schütz A. Test-Retest Reliability is not a Measure of Reliability or Stability: A Friendly Reminder. PsyArXiv. 2020;

23. Morken IS, Viddal KR, Ranum B, Wichstrøm L. Depression from preschool to adolescence – five faces of stability. J Child Psychol Psychiatry [Internet]. 2021;62:1000–9. Available from: <https://acamh.onlinelibrary.wiley.com/doi/10.1111/jcpp.13362>

24. Putnick DL, Bornstein MH. Measurement invariance conventions and reporting: The state of the art and future directions for psychological research. Dev Rev [Internet]. 2016;41:71–90. Available from: <https://linkinghub.elsevier.com/retrieve/pii/S0273229716300351>

25. Cheung GW, Rensvold RB. Structural Equation Modeling Evaluating Goodness-of-Fit Indexes for Testing Measurement Invariance. 2009 [cited 2022 Aug 31]; Available from: <https://www.tandfonline.com/action/journalInformation?journalCode=hsem20>

26. van de Schoot R, Lugtig P, Hox J. A checklist for testing measurement invariance. Eur J Dev Psychol. 2012;9:486–92.
